# Parametric Testing of Metasurface Stirrers for Metasurfaced Reverberation Chambers

**DOI:** 10.3390/s19040976

**Published:** 2019-02-25

**Authors:** Hengyi Sun, Changqing Gu, Zhuo Li, Qian Xu, Mengmeng Wei, Jiajia Song, Baijie Xu, Xiaohang Dong, Kuan Wang, Ferran Martín

**Affiliations:** 1 Key Laboratory of Radar Imaging and Microwave Photonics, Ministry of Education, College of Electronic and Information Engineering, Nanjing University of Aeronautics and Astronautics, Nanjing 211106, China; lizhuo@nuaa.edu.cn (Z.L.); emxu@foxmail.com (Q.X.); JJSNUAA@foxmail.com (J.S.); xbj12123@outlook.com (B.X.); dongxiaohang@hotmail.com (X.D.); kuanwang@nuaa.edu.cn (K.W.); 2 CIMITEC, Departament d’Enginyeria Electrònica, Universitat Autònoma de Barcelona, 08193 Bellaterra, Barcelona, Spain; hengyi.sun@e-campus.uab.cat; 3 College of Humanities & Social Development, Northwest A&F University, Yangling 712100, China; 18291892319@163.com

**Keywords:** metasurface, reverberation chamber, stirrer, field uniformity

## Abstract

In this paper, the correlation coefficients and the total scattering cross sections (TSCSs) for different types of metasurfaced stirrers and the traditional metallic stirrer, and the effects on field uniformity when such stirrers are used in reverberation chambers, are analyzed. Three metasurfaced stirrers are considered: A stirrer with two unit cells arranged alternatively (#1), a stirrer with two unit cells arranged in a chessboard-like manner (#2), and a stirrer with two unit cells in random arrangement (#3). From the correlation coefficient and TSCS results obtained in simulations, it follows that metasurfaced stirrer #1 is the best option. Field uniformity analysis of the resulting metasurface reverberation chambers (MRC) equipped with the different stirrers also supports this conclusion.

## 1. Introduction

In general, a reverberation chamber (RC) consists of a metal shielded chamber containing stirrers, antennas, a piece of equipment under test, as well as other devices. Formally, an RC can be defined as an electrically large, high quality factor (Q), multimode cavity that uses mode stirring to create changing boundary conditions in order to obtain a statistically uniform electromagnetic field [1,2]. RCs, also called mode-stirred chambers, are common test facilities widely used for electromagnetic compatibility (EMC) measurements, multipath environment characterization, electromagnetic immunity analysis, power radiation estimation, etc. [3].

In the past, many efforts have been focused on trying to optimize the performance of metallic stirrers in conventional RCs. Mainly through the application of empirical techniques and common sense, heuristic guides, which define and adopt many design criteria, have been proposed. For instance, stirrers should be greater than or equal to the wavelength of the lowest usable frequency (LUF), stirrers should be shaped such that a non-repeating field pattern is obtained in one rotation, etc. In this sense, references [3,4,5,6,7,8,9,10,11] provide some practical contributions to stirrer design and stirrer optimization, and there are examples of flexible stirrers that optimize RC performance by selecting the optimum angle of the bend [5,6], the position of the stirrer blades [7,8], or the independent number of the stirrer positions [9,10].

Despite the efforts directed towards stirrer optimization, certain limitations and constraints arise in real-life scenarios (e.g., large stirrers penalize the available test volume; complex stirrer shapes may hinder the design and implementation, thereby increasing costs). To date, few efforts have been concentrated on alternative stirring methods which may provide a solution to the previous limitative aspects. In this regard, the concept of a metasurfaced reverberation chamber (MRC), with stirrers based on metasurfaces, has been recently proposed [11] as an alternative to conventional RCs. Metasurfaces are the planar counterparts of metamaterials, consisting of a single-layer or few-layer stack of planar structures, which can be fabricated using lithography and nanoprinting methods, and are usually described by effective medium parameters at the macroscopic scale; see [11,12].

In [11], we used a 1-bit random coding metasurface to realize the MRC. The 1-bit random coding diffusion metasurface was designed in order to obtain all-direction backscattering under normal incidence. By rotating the metasurface stirrer, wave diffusion (at least in the transient electromagnetic field distribution) is ensured. Moreover, such a metasurface is not only a specific mode tuner, but also a phase wall with multiple boundary conditions. Thus, the mode structure changes at each stir state. The main conclusion in [11] was that although the field uniformity was only somewhat improved for the MRC with the rotating 1-bit coding diffusion metasurface (as compared to the traditional one), an extended MRC test zone was achieved due to the lack of mechanical stirrer. In [13], we analyzed the physical mechanism for increasing the number of modes, lowering the operating frequency, and improving the field uniformity in the reverberation chambers, and concluded that a designed sequential metasurface provided better stirring effect than the random one. After dealing with these studies, it is concluded that the coding metasurface stirrer can improve the uniformity of field distribution in the MRC, and can replace the traditional metallic stirrer. Moreover, using metasurface walls in the chamber is useful to increase the number of modes and to lower the useful frequency, especially for the metasurface with unit cells arranged alternatively [13].

In this work, we study and compare three different types of rotating metasurfaced stirrer (also including the mechanical metallic stirrer in the comparison) on the basis of their influence on RC performance. The key performance indicators are the correlation coefficients and total scattering cross section (TSCS).

### 1.1. Simulation Parameters Set Up

**Figure 1:**
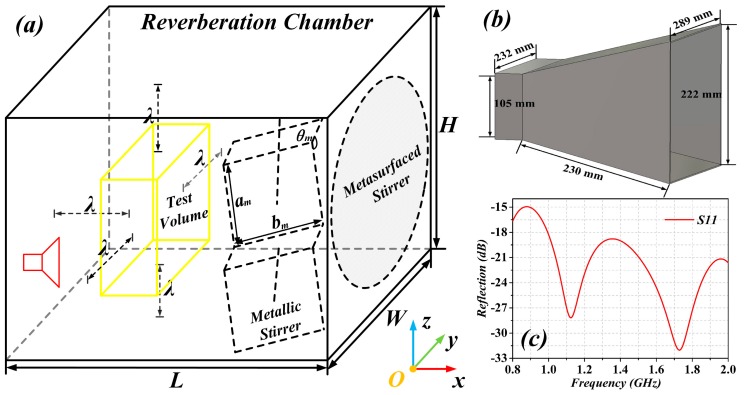
Schematic diagram of the reverberation chamber (RC) with metallic stirrer or with metasurfaced stirrer (**a**), three-dimensional view of the antenna (**b**), and its reflection coefficient (**c**). L = 1.2 m, W = 0.8 m and H = 1.2 m. Dimensions of the metallic stirrer were a_m_ = 0.3 m, b_m_ = 0.4 m, θ_m_ = 120° and the thickness of all the metallic blades was 2 mm. The diameter of the metasurfaced stirrer was 1.2 m, and the considered dielectric layer was 0.031 m thick, with dielectric constant *ε_r_* = 2.65 and loss tangent tanδ = 0.001.

The simulated RC was 1.2 m long, 0.8 m wide, and 1.2 m high, with a transmitting antenna as shown in Figure 1. The dimensions of the antenna and the simulation of the reflection coefficient in free space are also depicted in Figure 1 (the orientation of the antenna had no effect on simulation results, as it had been corroborated from independent results (not shown)). In our case, the distance of the boundary of test volume to the wall of the RC was ***λ*** = 0.375 m, where ***λ*** was the wavelength of the LUF (800 MHz, in our case, where the number of modes of the RC was above 100). It was at least half a meter from all other metallic surfaces and it remained the same for all the simulated configurations, for the sake of comparison, even though for some analyzed configurations it could have been greater.

**Figure 2:**
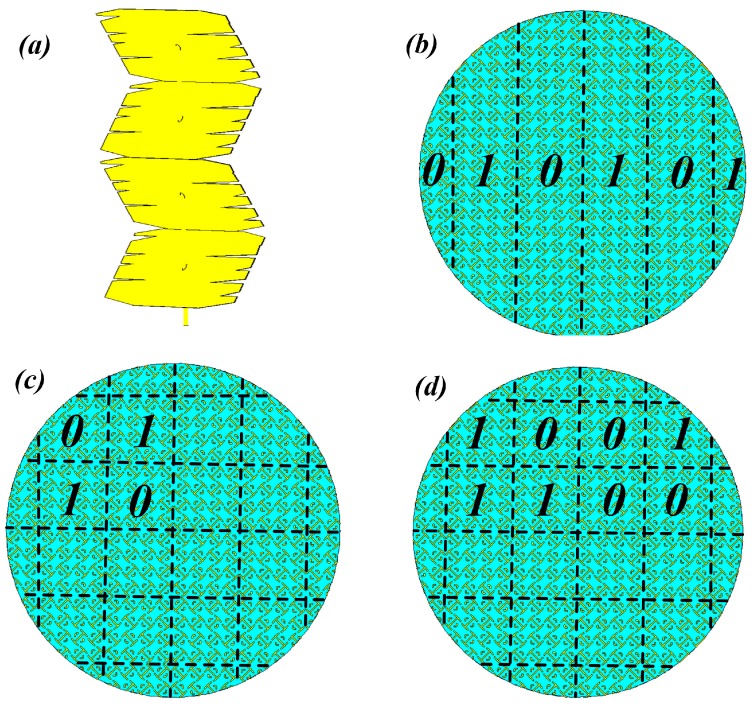
Metallic stirrer (**a**), periodic metasurfaced stirrer with coding sequences 0101…/0101... (**b**) and 0101…/1010… (**c**), and 1-bit random coding metasurfaced stirrer (**d**).

The considered metasurfaced stirrers in the present study were based on two types of unit cells, with “0” and “π” phase responses to mimic the “0” and “1” elements of the 1-bit digital coding metasurface (these can be controlled using existing digital technology [14]). The details of the unit cell are described in [11]. With such unit cells, we designed three different metasurfaced stirrers, which were compared with each other and with the metallic stirrer. The metasurfaced stirrers contained (i) two unit cells arranged alternatively (#1), (ii) two unit cells arranged in a chessboard-like manner (#2), and (iii) two unit cells in random arrangement (#3). By following the concept of coding metamaterial, case #1 was characterized by coding sequences 0101…/0101…, case #2 by coding sequences 0101…/1010…, and case #3 was the 1-bit random coding case (Figure 2).

**Figure 3:**
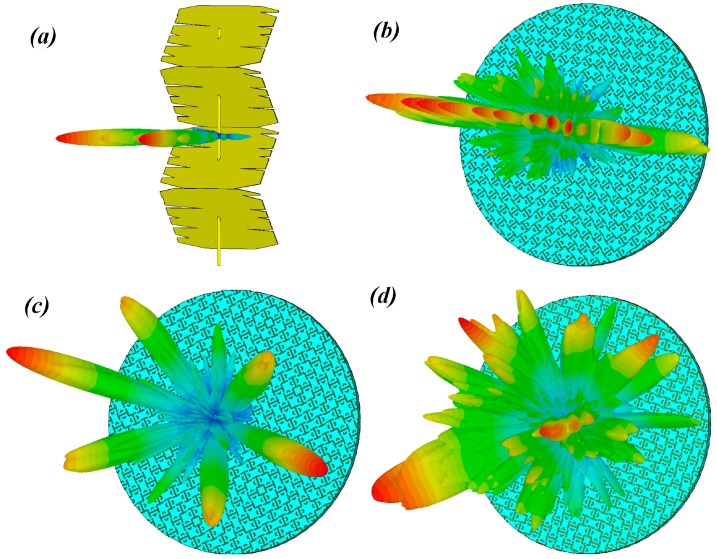
Simulated far-field pattern of the metallic stirrer (**a**) and metasurfaced stirrers (**b**) #1, (**c**) #2, and (**d**) #3 under normal incidence at 1 GHz (excitation with a plane wave is considered).

In order to analyze the scattering characteristic of the stirrers, we first simulated the far-field pattern of the stirrers by means of CST Microwave Studio^®^ (CST China Ltd. Nanjing, China). The different stirrers generated a diffuse far-field scattering pattern, as depicted in Figure 3. The pattern obtained by full-wave simulation was in agreement with the result of the optimization algorithm [14]. In the CST software, we set the maximum number of cells per wavelength to 20, both in near- and far-form model options.

### 1.2. Analysis of Simulation Results

**Figure 4:**
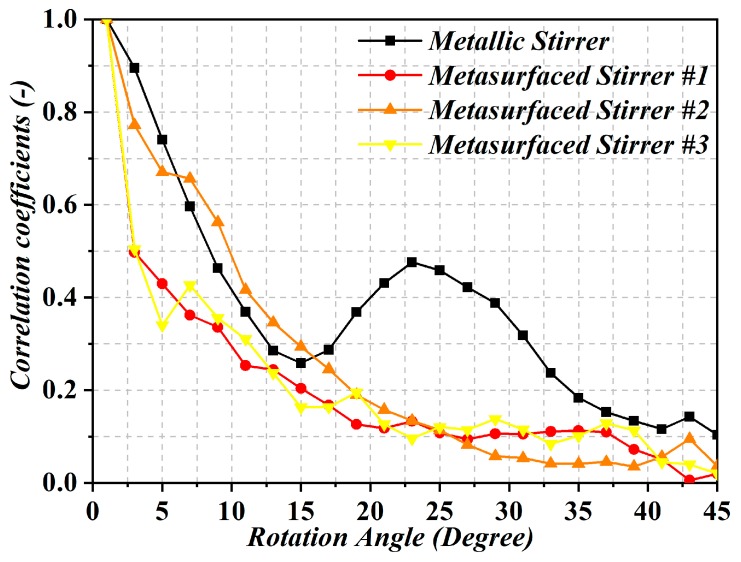
Correlation coefficients of the metallic and metasurfaced stirrers at 1 GHz.

To evaluate whether a stirrer provides independent field conditions for the different rotation positions, the correlation coefficient is the key parameter. It is assumed that uncorrelated stirrer positions yield independent field conditions [15]. Thus, the correlation coefficient between different three-dimensional far-field patterns of the metasurface corresponding to different rotation angles, as introduced in [11], was calculated. The correlation coefficients with the variations of the rotation angle from 0° to 45° in steps of 3° are shown in Figure 4. In the diagram, it can be observed that the correlation coefficients of stirrers #1 and #3 decrease sharply within 10 degrees of the rotation angle. This decrease in the correlation coefficient is indicative of the different electric field patterns between the different angles (due to changing boundary conditions), giving rise to an effective improvement of field uniformity.

The TSCS reveals an interesting criterion to quantify the target scattering ability. In the EMC regard, the TSCS of an RC provides an indication of the capability of the chamber to generate statistical field uniformity, through stirring efficiency [16,17,18,19,20]. To compute the TSCS in a reverberant environment, the commercial software CST Microwave Studio^®^ (CST China Ltd. Nanjing, China) was employed. Nevertheless, the theoretical foundations of TSCS are given as follows. The expression giving the mean value of the E-field *E_η_*(*t*) (*η* = *x*, *y*, *z*: Cartesian component) over the sources *α* (in our case, *α* = 1), probes *β* (in our case, *β* = 8) and over the various object locations *γ* (in our case, *γ* = 1) at time *t*, is as follows [18,21]:

(1) $${\langle {\langle E_{\eta }(t)\rangle }_{\gamma }^{2}\rangle }_{\alpha ,\beta }={\langle {\langle E_{\eta }(t=0)\rangle }_{\gamma }^{2}\rangle }_{\alpha ,\beta }e^{-t/\tau_{s}}.$$

The TSCS can be inferred from the scattering damping time *τ_s_*:

(2) $$TSCS=V/N\tau_{s}c,$$

where *V* is the volume of the chamber, *N* is the number of moving objects (in our case, *N* = 1) and *c* is the speed of light in vacuum. After rewriting (1), we can define the ratio *C*(*t*) as follows:

(3) $$C(t)=\frac{{\langle {\langle E_{\eta }(t)\rangle }_{\gamma }^{2}\rangle }_{\alpha ,\beta }}{{\langle \langle E_{\eta }^{2}(t=0)\rangle \rangle }_{\alpha ,\beta ,\gamma }}=e^{-t/\tau_{s}}.$$

Therefore, the scattering damping time *τ_s_* can be linearly derived from relations (1) and (3) according to:

(4) $$\tau_{s}=-\frac{t}{\ln\left[C(t)\right]},$$

where *C*(*t*) is inferred through the CST simulation.

**Figure 5:**
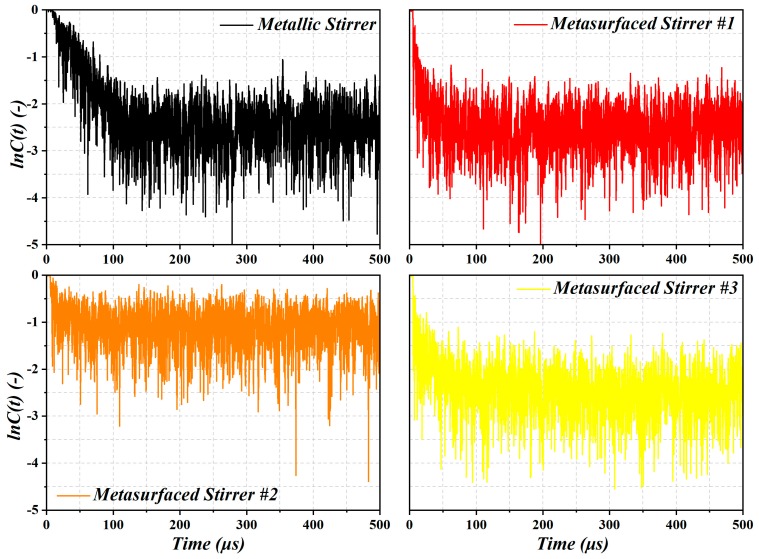
A comparison of the *C* ratio between the four stirrers.

In Figure 5, we plotted ln*C*(*t*) corresponding to the four stirrers (#1, #2, #3, and the metallic stirrer). After the estimation of the damping time (*τ_s_*), the TSCS was computed for the four stirrers (TSCS is inversely proportional to *τ_s_*, according to Expression 2). Note that for a high Q cavity, waves outperform many bounces within the cavity before they decay. Thus, the higher the Q value, the longer the *τ_s_* [22]. In other words, small *τ_s_* is associated with significant stirring. From the results of Figure 5, it can be clearly seen that ln*C*(*t*) of stirrers #1 and #3 reduces sharply, as compared to the metallic stirrer and stirrer #2. In other words, the stirrers #1 and #3 exhibited better TSCS and stirring efficiency. Note that *τ**_s_*** can be extracted from the least-square fit of the logarithm of *C*(*t*) in (3) [23,24], thus, the TSCS can be obtained using expression (2) (signals after 100 µs are the noise floor and are not used for the least-square fit). The stirrer was rotated through 12 stirrer positions (30 degrees/step). The values of TSCS for the four considered cases were found to be: 0.168 m^2^ (metallic stirrer), 0.195 m^2^ (metasurface stirrer #1), 0.123 m^2^ (metasurface stirrer #2), 0.189 m^2^ (metasurface stirrer #3). 

**Figure 6:**
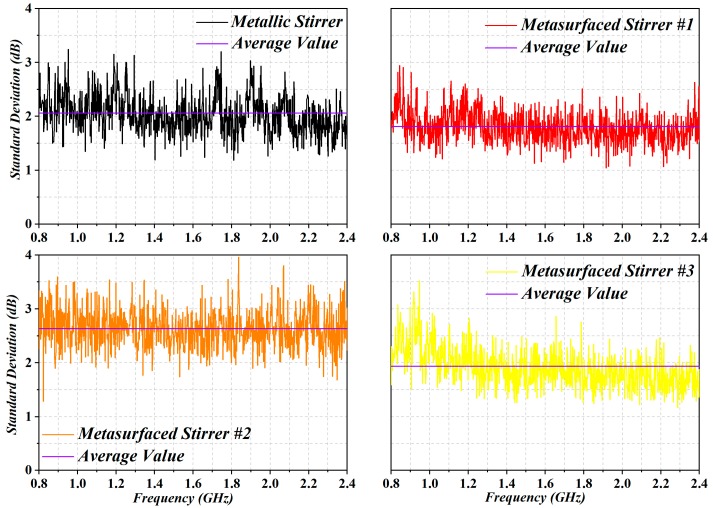
Standard deviation with respect to the mean value of maximum simulated obtained by each of the 12 steps, for a complete rotation of the stirrer.

To further verify our optimization results for the metasurfaced stirrers, some standard EMC tests [25] were carried out for the four stirrers. Particularly, the E-field components were recorded by isotropic probes placed on the eight corners of the test volume. According to [25], and taking into account the LUF of our metasurfaced reverberation chamber configuration and the studied bandwidth, the simulations were repeated for 12 steps of the stirrer rotation. The uniformity and the isotropy of the E-field are defined as the standard deviation with respect to the mean value of the maximum measures obtained for each of the eight probes and for a complete rotation of the stirrer. The results are shown in Figure 6. In the figure, the acceptable limits for field uniformity decreed in [25] are within 3 dB above 400 MHz. We plotted the standard deviation calculated from the mean value of the maximum E-field by giving equal weight to each component. The average values of the standard deviation were 2.06 dB, 1.81 dB, 2.63 dB, and 1.93 dB for the metallic stirrer and metasurfaced stirrers (#1, #2, #3), respectively. Thus, metasurfaced stirrer #1 provided a greater scattering efficiency compared to the others. We note that metasurfaced stirrer #1 respected the tolerance constraint, in accordance with the results of correlation coefficient and TSCS. From all these results, we can conclude that metasurfaced stirrer #1 exhibited much better stirring efficiency.

## 2. Conclusions

In this paper, we have analyzed the effects of introducing different types of metasurface stirrers in reverberation chambers, compared to the traditional metallic stirrer. Three metasurface stirrers have been studied: one with two unit cells arranged alternatively (#1), another one with two unit cells arranged in a chessboard-like manner (#2), and a third one with two unit cells in random arrangement (#3). From the correlation coefficient and TSCS results obtained, we can conclude that the field uniformity is somehow improved for the RC with metasurfaced stirrer #1. Since this stirrer is comprised of a metasurface with negligible volume (as compared to a metallic stirrer), the test volume of the RC is significantly extended. The volume extension is dependent on the dimensions of the mechanical stirrer.
